# Thiamine and its phosphate esters in relation to cardiometabolic risk factors in Saudi Arabs

**DOI:** 10.1186/2047-783X-18-32

**Published:** 2013-09-23

**Authors:** Nasser M Al-Daghri, Omar S Al-Attas, Khalid M Alkharfy, Majed S Alokail, Sherif H Abd-Alrahman, Shaun Sabico

**Affiliations:** 1 Biomarkers Research Program, College of Science, King Saud University, Riyadh 11451, Kingdom of Saudi Arabia; 2 Prince Mutaib Chair for Biomarkers of Osteoporosis, King Saud University, Riyadh 11451, Kingdom of Saudi Arabia; 3 Center of Excellence in Biotechnology Research Center, King Saud University, Riyadh 11451, Kingdom of Saudi Arabia; 4 Clinical Pharmacy Department, College of Pharmacy, King Saud University, Riyadh 11451, Kingdom of Saudi Arabia

**Keywords:** Arabs, Metabolic syndrome, Thiamine

## Abstract

**Background:**

Thiamine deficiency has suggested to be linked to several insulin-resistance complications. In this study, we aim to associate circulating thiamine levels among cardiometabolic parameters in an Arab cohort using a simple, sensitive, rapid and selective high-performance liquid chromatography (HPLC) method that has recently been developed.

**Methods:**

A total of 236 randomly selected, consenting Saudi adult participants (166 males and 70 females) were recruited and screened for the presence of the metabolic syndrome (MetS) using the modified National Cholesterol Education Program–Adult Treatment Panel III definition. Blood thiamine and its derivatives were quantified using HPLC.

**Results:**

A total of 140 participants (53.9%) had MetS. The levels of thiamine and its derivatives of those with MetS were not significantly different from those without. However, hypertensive subjects had significantly higher urinary thiamine (*P* = 0.03) as well as significantly lower levels of thiamine diphosphate (TDP) (*P* = 0.01) and total thiamine (*P* = 0.02) than the normotensive subjects, even after adjusting for age and body mass index (BMI). Furthermore, age- and BMI-matched participants with elevated blood glucose levels had significantly lower levels of thiamine monophosphate (*P* = 0.020), TDP (*P* < 0.001) and total thiamine (*P* < 0.001) and significantly elevated levels of urinary thiamine (*P* = 0.005) compared to normoglycemic participants.

**Conclusions:**

Low thiamine levels are associated with elevated blood glucose and hypertension in Saudi adults. Determination of thiamine status may be considered and corrected among patients with, or at high risk for, MetS, but the question whether thiamine deficiency correction translates to improved cardiometabolic status needs further longitudinal investigation.

## Background

Thiamine (vitamin B1) is an essential water-soluble vitamin with major functions in carbohydrate metabolism. It is phosphorylated by thiamine kinase, and the major fraction of tissue thiamine is represented by thiamine diphosphate (TDP) [1]. This active form functions as a cofactor for both pyruvate (PDHC) and α-ketoglutarate dehydrogenase complexes (KDHCs). Studies have shown that decreased activity of PDHC, KDHC or succinate dehydrogenase secondary to thiamine deficiency results in acetyl-coenzyme A and energy deficits in brain, muscle and other tissues [2-5].

The TDP concentration in erythrocytes has been observed to be a good indicator of body stores because it depletes at a rate similar to TDP levels found in major organs of the body [6]. Studies have suggested that erythrocyte TDP concentrations determined by both the apoenzyme recombination technique and high-performance liquid chromatography (HPLC) are more sensitive indices of thiamine status than measurement of erythrocyte transketolase activity [7,8]. The use of HPLC for the direct determination of TDP, however, has clear advantages in terms of sensitivity, specificity, precision and robustness [7-9].

HPLC methods for the determination of thiamine and its esters in blood have been documented [10]. We have previously developed a step-gradient HPLC method to overcome the weaknesses of previously reported thiamine assays in terms of speed and accuracy for baseline separation of thiamine compounds using whole blood instead of washed erythrocytes and a simpler mobile phase [11]. In the present study, we aim to determine associations of thiamine and its derivatives with cardiometabolic parameters in a Saudi cohort with or without metabolic syndrome (MetS) using our method of thiamine quantification. The study will be one of the first to shed light on the association of thiamine with MetS parameters among the Saudi Arab ethnicity, in which MetS manifestations and other chronic noncommunicable diseases are highly prevalent [12,13].

## Methods

### Participants

Samples were taken from 236 randomly selected, healthy, consenting Saudi adult participants (166 males and 70 females) who were part of the Biomarkers Screening in Riyadh (BSR), a capital-wide survey spanning all primary care centers in Riyadh initiated by the Biomarkers Research Program (BRP) of King Saud University (KSU) and the Ministry of Health. In brief, BSR was launched to identify and employ novel biomarkers of chronic noncommunicable diseases, including diabetes mellitus (DM), cardiovascular disease (CVD), hypertension and obesity, among consenting and randomly recruited Saudis. Ethical approval was obtained from the Ethics Committee of the College of Science Research Center of KSU, Riyadh, Saudi Arabia [12]. They were screened for the presence of MetS based on the definition of the modified National Cholesterol Education Program–Adult Treatment Panel III.

### Reagents

A certified thiamine hydrochloride standard was obtained from Dr Ehrenstorfer GmbH (Augsburg, Germany). Thiamine monophosphate (TMP), TDP, potassium ferricyanide, trichloroacetic acid (TCA) and sodium hydroxide were obtained from Sigma Chemical Company (St Louis, MO, USA). HPLC-grade acetonitrile and methanol were purchased from VWR International (VWR International, Lutterworth, UK).

### Urine sample preparation

Urine samples were extracted according to a previously described method [14]. In summary, samples were thawed in the dark at room temperature. After brief mixing, 0.1 ml of urine samples were transferred to a 2-ml amber glass tube and 0.9 ml of 0.01 M HCl was added, followed by vortex mixing. The resulting solution was then filtered through a Millipore polytetrafluoroethylene 0.45-μm/25-mm filter (EMD Millipore, Billerica, MA, USA) into light-protected vials and placed in the autosampler tray of the HPLC for analysis.

### Blood sample preparation

Fasting blood was drawn (about 10 ml), centrifuged and processed on the same day the participants submitted their consent forms. Both whole blood and serum were placed in plain polystyrene tubes. Collected fasting blood samples were delivered to BRP for storage at −20°C. On the day of analysis, blood samples were thawed at room temperature. An aliquot of 300 μl of the sample was added to 50 μl of TCA (TCA concentration = 50%). This aliquot was mixed using a vortex shaker for 1 min and centrifuged at 6,000 rpm/10 min. The resulting supernatant (250 μl) was collected and transferred into clean 300 μl microautosampler vials. A freshly prepared derivatizing reagent (0.2% potassium ferricyanide prepared in 15% sodium hydroxide) will subsequently be added (20 μl) to the samples before injection.

### Instrumental analysis

The BRP, College of Science, KSU, Riyadh, Saudi Arabia, has an HPLC system (Shimadzu Corp, Kyoto, Japan) that is fully equipped with a model series (LC-10ADVP pump, DGU-14A degasser, SIL-10ADVP autosampler, SPD-M20A UV/VIS detector, RF-10AXL fluorescence detector and SCL-10A system controller; Shimadzu Corp). System control and data analysis were carried out using LCsolution software (Shimadzu Corp). Separation of these analytes has been done on a Symmetry C18 column (5 μm; 250 mm × 4.6 mm; Waters Corp, Milford, MA, USA). The separation was carried out using a gradient elution procedure. Mobile phase A (30 mM sodium dihydrogen phosphate buffer adjusted to pH 4.5 and acetonitrile; 94:6 vol/vol ratio) and mobile phase B was 30 mmol/L sodium dihydrogen phosphate buffer (pH 4.5) and acetonitrile (70:30 vol/vol) ratios were linearly changed as follows: 0 to 3 min, 0%:30% mobile phase B; 3 to 5 min, 30%:90% mobile phase B; 5 to 9 min, 90% mobile phase B; 9 to 10 min, 90%:0% mobile phase B; and 10 to 20 min, 0.0% mobile phase B. The total run time was 20 min at a flow rate of 0.7 ml/min. The eluent was monitored by a diode array detector and detection wavelength was set at 254 nm. Detection of thiamine was observed after precolumn derivatization at the excitation and emission wavelengths of 365 and 435 nm, respectively, with an injection volume of 50 μl.

Chromatographic conditions, as well as analytical validation, which includes linearity and detection limit, recovery, precision and accuracy, have been described previously [11]. In brief, the standard calibration curves showed good linearity for thiamine at 0, 25, 50, 100, 150 and 200 ng/ml and 0, 10, 20, 40 and 80 ng/ml for both TMP and TDP, respectively. Correlation coefficient values are *r* > 0.98 for thiamine, *r* > 0.97 for TMP and *r* > 0.99 for TDP. Recovery percentages were 93.0% ± 4.0%, 97.0% ± 5.4% and 91.0% ± 3.4% for the three analytes, respectively.

### Statistical analysis

Data were analyzed using the SPSS version 16.5 software (SPSS, Chicago, IL, USA). Continuous variables are presented as means ± standard deviation, and variables exhibiting non-Gaussian distribution were normalized prior to analysis. Independent *t*-tests were used to compare groups between those with and without MetS. The same test was employed between normotensive versus hypertensive and normoglycemic versus hyperglycemic, with adjustments for age and body mass index (BMI). Regression analysis was done to determine associations of interest among variables. *P*-value significance was set at <0.05.

## Results

### Characteristics of participants with or without MetS

Table 1 shows the general characteristics and unadjusted comparisons of participants with or without MetS. As expected, those who harbor MetS had significantly higher anthropometric measures, including BMI (*P* < 0.001), waist circumference (*P* < 0.001) and hip circumference (*P* < 0.001), as well as sagittal abdominal diameter (SAD) (*P* < 0.001) and systolic blood pressure (*P* = 0.011) than those without MetS. Among the biochemical parameters, those with MetS also had significantly higher levels of fasting blood glucose (*P* = 0.010) and triglycerides (*P* = 0.025), as well as significantly lower urinary albumin levels (*P* = 0.030), than those without MetS. The rest of the parameters, including thiamine and its derivatives, were comparable.

**Table 1:** **General characteristics of participants with or without metabolic syndrome**^**a**^

**Characteristics**	**Control**	**MetS**	***P*-value**
*N*	96	140	
Gender (M/F)	69/27	97/43	
Age (yr)	49.4 ± 16.5	53.7 ± 13.3	0.03
BMI (kg/m^2^)	27.6 ± 6.0	31.3 ± 6.3	< 0.001
Waist circumference (cm)	81.5 ± 23.8	106.1 ± 12.5	< 0.001
Hip circumference (cm)	91.5 ± 26.8	112.1 ± 15.7	< 0.001
Sagittal abdominal diameter (cm)	22.3 ± 9.2	26.7 ± 7.1	< 0.001
Systolic blood pressure (mmHg)	124.3 ± 16.8	130.0 ± 14.3	0.01
Diastolic blood pressure (mmHg)	78.8 ± 10.5	81.0 ± 8.5	0.10
Triglycerides (mmol/L)	1.6 ± 0.13	1.9 ± 0.11	0.02
Total cholesterol (mmol/L)	5.0 ± 1.1	5.3 ± 1.2	0.12
LDL cholesterol (mmol/L)	3.5 ± 1.0	3.7 ± 1.0	0.18
HDL cholesterol (mmol/L)	0.70 ± 0.29	0.63 ± 0.24	0.07
Glucose (mmol/L)	7.4 ± 1.6	9.0 ± 1.5	0.01
Thiamine (ng/ml)	3.3 ± 0.13	3.2 ± 0.16	0.88
TMP (ng/ml)	2.1 ± 1.4	2.3 ± 1.5	0.19
TDP (ng/ml)	27.4 ± 1.8	22.3 ± 2.0	0.06
Total thiamine (ng/ml)	35.0 ± 1.8	31.3 ± 2.3	0.20
Thiamine (urine) (μg/ml)	938.0 ± 170.8	947.1 ± 207.4	0.94
Albumin (serum) (g/L)	47.8 ± 5.6	46.7 ± 6.4	0.31
Albumin (urine) (mg/L)	23.8 ± 4.1	16.7 ± 3.3	0.03
Serum creatinine (mmol/L)	78.6 ± 1.2	81.7 ± 1.5	0.52
Urinary creatinine (mmol/L)	11,966.8 ± 1,008.7	9,662.9 ± 1,393.6	0.07
Calcium (mmol/L)	2.6 ± 0.51	2.6 ± 0.50	0.78
Phosphorous (mmol/L)	1.3 ± 0.36	1.4 ± 0.43	0.25

*Note*: Data represent means ± standard deviation. Independent *t*-tests were done for two groups. *P* ≤ 0.05 was used as the significance level. ^a^*BMI* body mass index, *HDL* high-density lipoprotein, *LDL* low-density lipoprotein, *MetS* metabolic syndrome, *TDP* thiamine diphosphate, *TMP* thiamine monophosphate.

### Differences in thiamine levels based on cardiometabolic parameters

We then compared parameters based on the presence of cardiometabolic risk factors. Table 2 shows age- and BMI-adjusted differences in the metabolic parameters of participants with or without hypertension. The hypertensive individuals had significantly higher levels of fasting blood glucose (*P* = 0.04), triglycerides (*P* = 0.02), phosphorous (*P* = 0.04) and urinary thiamine (*P* = 0.03), as well as significantly lower levels of TDP (*P* = 0.01) and total thiamine (*P* = 0.02), than the normotensive participants.

**Table 2:** **Age- and body mass index–adjusted comparisons between normotensive and hypertensive participants**^**a**^

**Characteristics**	**Normotensive**	**Hypertensive**	***P*-value**
*N*	117	119	
Gender (M/F)	81/36	85/34	
Systolic blood pressure (mmHg)	114.76 ± 8.85	137.67 ± 11.5	< 0.001
Diastolic blood pressure (mmHg)	73.6 ± 6.99	85.25 ± 7.6	< 0.001
Triglycerides (mmol/L)	1.3 ± 0.03	1.4 ± 0.03	0.02
Total cholesterol (mmol/L)	5.0 ± 1.0	5.4 ± 1.2	0.12
LDL cholesterol (mmol/L)	3.5 ± 0.10	3.7 ± 0.10	0.20
HDL cholesterol (mmol/L)	0.62 ± 0.03	0.70 ± 0.03	0.08
Glucose (mmol/L)	8.7 ± 0.41	9.9 ± 0.41	0.04
Thiamine (ng/ml)	3.4 ± 0.05	3.1 ± 0.05	0.30
TMP (ng/ml)	2.2 ± 0.04	2.3 ± 0.04	0.51
TDP (ng/ml)	27.7 ± 1.1	21.2 ± 1.1	0.01
Total thiamine (ng/ml)	35.0 ± 1.1	27.4 ± 1.1	0.02
Thiamine (urine) (μg/ml)	835.6 ± 2.0	1,049.6 ± 2.0	0.03
Albumin (serum) (g/L)	46.8 ± 0.73	47.2 ± 0.80	0.75
Albumin (urine) (mg/L)	17.3 ± 1.1	21.0 ± 1.2	0.30
Serum creatinine (mmol/L)	80.7 ± 0.20	80.6 ± 0.20	0.99
Urinary creatinine (mmol/L)	9,748.2 ± 20.0	10,774.4 ± 21.0	0.44
Calcium (mmol/L)	2.5 ± 0.47	2.7 ± 0.50	0.07
Phosphorous (mmol/L)	1.3 ± 0.41	1.5 ± 0.40	0.04

*Note*: Data represent means ± standard deviation. Level of significance was set at *P* ≤ 0.05. ^a^*HDL* high-density lipoprotein, *LDL* low-density lipoprotein, *TDP* thiamine diphosphate, *TMP* thiamine monophosphate.

Table 3 shows age- and BMI-adjusted differences in the metabolic parameters of participants with or without elevated blood glucose. Those with elevated blood glucose had significantly lower levels of high-density lipoprotein (HDL) cholesterol (*P* = 0.0055), TMP (*P* = 0.020), TDP (*P* < 0.001) and total thiamine (*P* < 0.001), as well as significantly elevated levels of urinary thiamine (*P* = 0.005), than normoglycemic participants.

**Table 3:** **Age- and body mass index–adjusted comparisons between normoglycemic and hyperglycemic participants**^**a**^

**Characteristics**	**Normoglycemic**	**Hyperglycemic**	***P*-value**
N	65	171	
Gender (M/F)	34/31	135/36	
Glucose (mmol/L)	4.6 ± 1.3	9.7 ± 1.4	< 0.001
Triglycerides (mmol/L)	1.6 ± 0.06	1.9 ± 0.03	0.23
Total cholesterol (mmol/L)	5.2 ± 0.20	5.1 ± 0.10	0.94
LDL cholesterol (mmol/L)	3.6 ± 1.0	3.6 ± 1.0	0.91
HDL cholesterol (mmol/L)	0.76 ± 0.04	0.62 ± 0.03	0.005
Thiamine (ng/ml)	3.5 ± 0.06	3.1 ± 0.04	0.14
TMP (ng/ml)	2.5 ± 0.06	2.1 ± 0.04	0.020
TDP (ng/ml)	36.2 ± 1.1	21.4 ± 1.0	< 0.001
Total thiamine (ng/ml)	41.7 ± 1.1	27.7 ± 1.0	< 0.001
Thiamine (urine) (μg/ml)	685.1 ± 3.3	1040.7 ± 2.0	0.005
Albumin (serum) (g/L)	46.2 ± 4.6	47.3 ± 6.4	0.18
Albumin (urine) (mg/L)	18.3 ± 1.2	19.2 ± 1.1	0.93
Serum creatinine (mmol/L)	78.8 ± 1.2	81.3 ± 2.0	0.68
Urine creatinine (mmol/L)	10,140.5 ± 43.9	10,261.7 ± 25.6	0.93
Calcium (mmol/L)	2.5 ± 0.08	2.6 ± 0.05	0.68
Phosphorous (mmol/L)	1.5 ± 0.07	1.4 ± 0.04	0.21

*Note*: Data represent means ± standard deviation. Level of significance was set at *P* ≤ 0.05. ^a^*HDL* high-density lipoprotein, *LDL* low-density lipoprotein, *TDP* thiamine diphosphate, *TMP* thiamine monophosphate.

**Figure 1:**
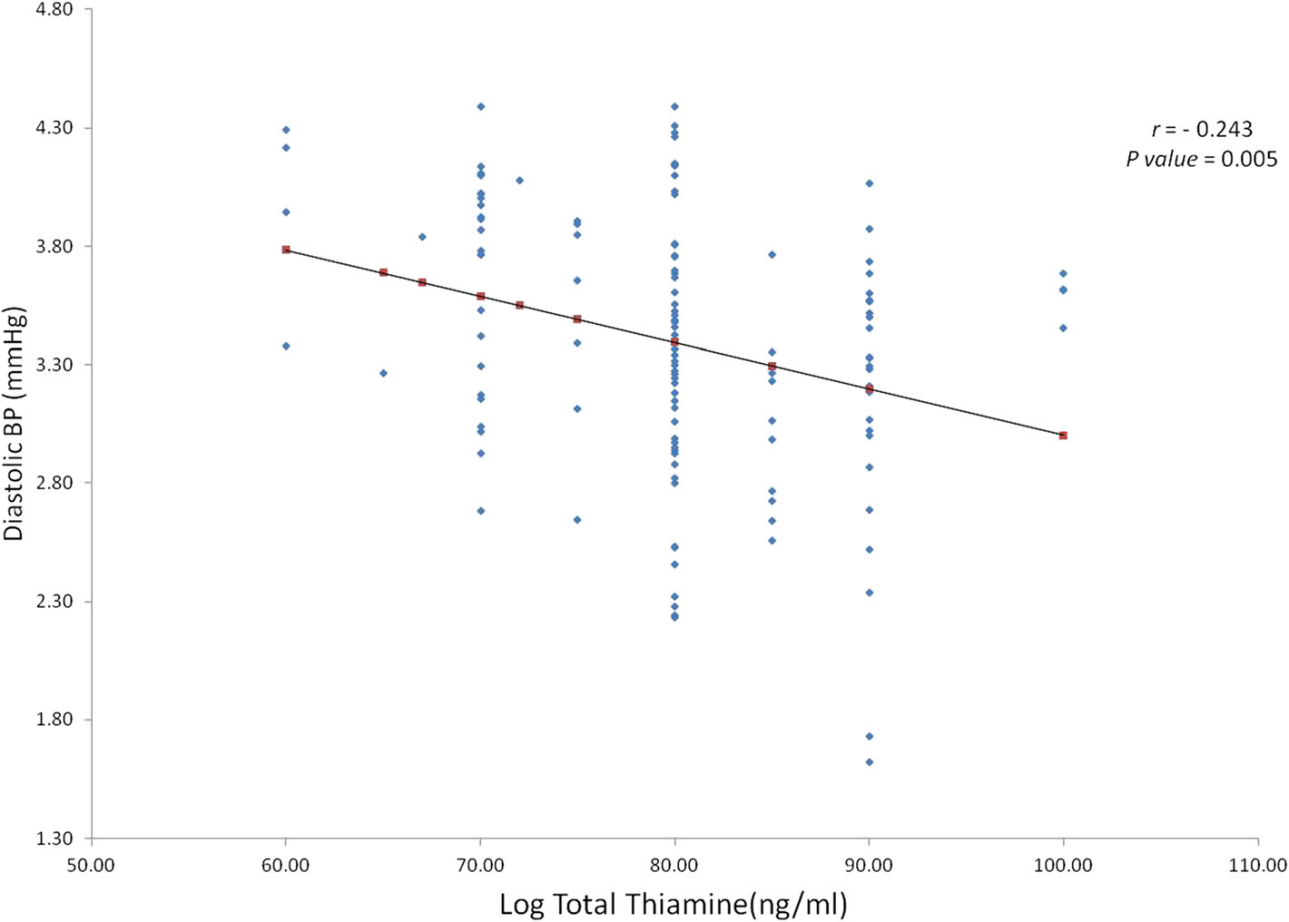
Inverse association between total thiamine and diastolic blood pressure.

Comparisons of those with versus those without dyslipidemia, as well as those with elevated BMI, were also done, but they did not show significant differences in both serum and urinary thiamine levels. Figure 1 shows the modest but significant inverse association of serum total thiamine to diastolic blood pressure (*R* = −0.24, *P* value = 0.005). Total thiamine was also associated with age (*R* = −0.16, *P* = 0.04); glucose (*R* = − 0.32, *P* < 0.001) and HDL cholesterol (*R* = −0.17, *P* = 0.04) (not shown in tables).

## Discussion

The present cross-sectional study is the first to highlight the significant inverse associations of total thiamine to cardiometabolic parameters in a Saudi Arab cohort with or without the manifestations of MetS. MetS, being a complex disorder of interconnected risk factors, has consistently been tied to increased risk of atherosclerotic CD and diabetes mellitus type 2 (DMT2) [15]. Several recent studies done in Nigeria highlighted that water-soluble vitamins, including thiamine, and antioxidants are significantly lower in people with MetS [15,16]. Thiamine deficiency has been implicated in diabetic complications and other vascular diseases [17,18]. Its relation to blood pressure can be explained through its renal excretion. Being a water-soluble vitamin, it is normally salvaged by reabsorption from the glomerular filtrate [19], which is altered in the presence of an underlying renal pathology, a common cause of hypertension.

Thiamine deficiency has also been noted to be common among Saudi patients with DMT2 [11], and high-dose thiamine therapy has been shown to improve the condition of diabetic patients with renal complications, making it a promising adjuvant therapy for DMT2 patients with known thiamine deficiency [20]. The association of thiamine levels with glucose has been documented elsewhere, but not in select lipid parameters such as HDL cholesterol [21]. Animal studies, however, show correction of triglycerides and total and LDL cholesterol after high-dose thiamine supplementation, still with the exception of HDL cholesterol, but nevertheless suggesting possible cardioprotective effects, probably secondary to food intake suppression and hexosamine pathway signaling [22-24].

The authors acknowledge several limitations of this study. The results of the present study are at most suggestive, because the study design is cross-sectional. Furthermore, other confounders, such as physical activity and diet, were not taken into consideration, so we cannot rule out possible residual confounders with the analysis done. Nevertheless, this study is the first to document the association of circulating thiamine to cardiometabolic risk factors in an Arab cohort, suggesting that micronutrient deficiencies such as thiamine deficiency have cardiovascular implications if not corrected. Whether the present findings apply to Arab children also needs to be investigated because metabolic traits are highly heritable in this particular population [25]. Further studies that are interventional in design should be considered to validate findings.

## Conclusions

Circulating thiamine and its derivatives are associated with blood glucose and blood pressure among Saudi Arabs with manifestations of MetS. Determination of thiamine status and correction thereof among patients with, or at high risk for, MetS may be considered. Longitudinal studies are needed to determine whether correction of thiamine deficiency can confer a favorable metabolic profile among those at risk for developing DMT2.

## Competing interests

The authors declare no competing interests.

## Authors’ contributions

NMA and OSA conceptualized the study. KMA, MSA and SHA performed sample, as well as data collection, analysis and interpretation. SS wrote the manuscript. All authors approved the final version of the manuscript.
